# Telemedicine and artificial intelligence in family medicine practice: a systematic review and meta-analysis of barriers and enablers in routine primary care

**DOI:** 10.3389/fdgth.2026.1843244

**Published:** 2026-08-10

**Authors:** Mohammed Nasser Albarqi

**Affiliations:** Department of Family and Community Medicine, College of Medicine, King Faisal University, Al-Ahsa, Saudi Arabia

**Keywords:** artificial intelligence, clinical decision support, digital health, family practice, implementation barriers, primary care, systematic review, telemedicine

## Abstract

**Introduction:**

Telemedicine and artificial intelligence (AI) are reshaping healthcare delivery, yet their integration into routine family practice remains inconsistent. This systematic review and meta-analysis aimed to identify barriers and enablers influencing the adoption of telemedicine and AI in primary care and to quantify their effects on clinical and operational outcomes.

**Methods:**

This review followed PRISMA guidelines and was registered with PROSPERO (CRD420251029675). Six databases—PubMed, Scopus, Web of Science, Cochrane Library, CINAHL, and IEEE Xplore—were searched from inception to March 15, 2025. Eligible studies were randomized controlled trials examining telemedicine or AI implementation in family practice or primary care settings. Data extraction covered study characteristics, intervention type, outcomes, and reported barriers and enablers. Risk of bias was assessed using the Cochrane Risk of Bias 2 tool. Narrative synthesis, thematic analysis, and random-effects meta-analysis were conducted where appropriate.

**Results:**

Thirteen randomized controlled trials involving more than 29,000 participants were included. Telemedicine interventions showed favorable study-level effects on chronic disease management, access to care, and patient satisfaction, although the pooled clinical effect across ten trials did not reach statistical significance. AI applications significantly improved diagnostic accuracy and clinical decision-making, with a pooled log odds ratio of 0.73 (95% CI: 0.46–1.00). Key enablers included clinician engagement, reliable digital infrastructure, leadership support, training, and workflow integration. Major barriers included technological interoperability, clinician skepticism, patient digital literacy limitations, privacy concerns, and uncertain reimbursement or governance frameworks.

**Discussion:**

Telemedicine and AI have promising roles in strengthening family practice, particularly for access, chronic disease management, diagnostic support, and decision-making. Sustainable implementation requires attention to technological, organizational, regulatory, and human factors, alongside targeted training, policy support, and integration into routine primary care workflows.

## 1 Introduction

The integration of telemedicine and artificial intelligence (AI) into family practice is rapidly reshaping the landscape of primary healthcare delivery. Driven by the dual imperatives of improving healthcare access and optimizing clinical decision-making, these technologies have emerged as pivotal tools in addressing the demands of modern healthcare systems worldwide (34). As health systems face growing burdens from aging populations, chronic diseases, and the persistent inequities in access to care, telemedicine and AI offer promising avenues for enhancing the efficiency, reach, and personalization of care in routine practice settings, especially within family medicine—the cornerstone of primary care delivery (10, 32).

Telemedicine, defined broadly as the remote delivery of healthcare services using telecommunications technologies, has evolved from a niche service to a mainstream component of healthcare in many countries (26). Its benefits include reducing geographical barriers, decreasing patient travel time, and enabling real-time consultations, especially in underserved or rural areas (24). The COVID-19 pandemic further catalyzed the widespread adoption of telemedicine, demonstrating its potential to maintain continuity of care during times of restricted mobility and heightened demand for healthcare resources (59). Concurrently, AI technologies—ranging from machine learning algorithms to natural language processing and predictive analytics—are being increasingly embedded within electronic health records (EHRs), diagnostic tools, and clinical decision support systems (CDSS), offering clinicians robust data-driven insights that can improve diagnostic accuracy, risk stratification, and care planning (7, 20).

In the context of family practice, these technological innovations align closely with the principles of person-centered, continuous, and comprehensive care. Family physicians manage a broad spectrum of health issues across the lifespan and often serve as the first point of contact within the healthcare system (38). The incorporation of telemedicine and AI in this setting has the potential to enhance patient engagement, streamline administrative processes, and support evidence-informed clinical decisions (30). For example, AI-enhanced risk prediction models can assist family physicians in early identification of patients at risk for chronic conditions such as diabetes or cardiovascular disease, while teleconsultations can improve follow-up adherence and patient satisfaction (1).

Despite these potential benefits, the integration of telemedicine and AI into routine family practice remains fraught with challenges. These barriers span multiple domains, including technological, organizational, regulatory, and human factors. On the technological front, issues such as interoperability of systems, data privacy and security, and the digital divide continue to hinder seamless implementation (12). Clinicians may face difficulties in adapting to new workflows, concerns about loss of patient rapport during virtual encounters, and a lack of confidence in AI-generated recommendations (2, 49). Organizationally, the absence of standardized implementation frameworks, insufficient reimbursement models, and variable training in digital health competencies further complicate integration efforts (61). Regulatory and ethical challenges, including unclear liability in AI-supported decisions and concerns about algorithmic bias, also raise important considerations for safe and equitable use (8, 9).

Conversely, a growing body of literature highlights several enablers that facilitate successful adoption of telemedicine and AI in family practice. Key facilitators include supportive leadership, availability of technical infrastructure, integration with existing clinical workflows, and stakeholder engagement throughout the implementation process (4). Training and capacity building, particularly in digital literacy and AI ethics, play a critical role in promoting acceptance and sustained use among healthcare providers (23). Moreover, policy-level incentives, such as telehealth reimbursement schemes and national digital health strategies, have been shown to foster more favorable environments for innovation uptake (5, 21, 48).

Despite the increasing proliferation of research on telemedicine and AI in healthcare, there remains a lack of synthesis specifically focused on their integration within family practice settings. Most existing reviews either examine telemedicine or AI in isolation, or focus on hospital-based or specialty care contexts, thereby neglecting the unique challenges and opportunities present in primary care environments (33). Furthermore, while individual studies often describe implementation experiences, a systematic appraisal of the collective evidence on both barriers and enablers is lacking (36). This gap in the literature limits the ability of policymakers, clinicians, and health system planners to draw informed conclusions about the conditions necessary for successful integration in family practice (47).

### 1.1 Aim of the study

The aim of this systematic review and meta-analysis is to examine the integration of telemedicine and artificial intelligence (AI) in family practice by identifying, synthesizing, and quantifying the barriers and enablers affecting their implementation in routine care. This study seeks to evaluate the extent to which these digital innovations are being adopted in primary care settings, assess their impact on clinical and operational outcomes, and provide evidence-based insights to inform future policy, practice, and research.

### 1.2 Research question

What are the key barriers and enablers influencing the integration of telemedicine and artificial intelligence in routine family practice care, and what is the quantitative impact of these technologies on primary care delivery?

## 2 Materials and methods

### 2.1 Search strategy and selection criteria

This systematic review was conducted in accordance with the Preferred Reporting Items for Systematic Reviews and Meta-Analyses (PRISMA) guidelines to ensure methodological rigor and transparency in reporting. A comprehensive research protocol was developed and prospectively registered with the International Prospective Register of Systematic Reviews (PROSPERO) under the registration number CRD420251029675, reflecting our commitment to systematic integrity and scholarly accountability.

To ensure the identification of a wide spectrum of relevant literature, a thorough search strategy was employed across multiple leading databases, including PubMed, Scopus, Web of Science, CINAHL, IEEE Xplore, and the Cochrane Library. The final database search was executed on March 15, 2025, encompassing publications from inception through the search date. The strategy utilized a combination of Medical Subject Headings (MeSH) and keyword terms to capture the breadth of research involving telemedicine, artificial intelligence, family medicine, and the factors influencing their integration into routine care.

Search terms were iteratively refined in consultation with an academic librarian and domain experts to maximize sensitivity and specificity. Key terms included but were not limited to: “telemedicine,” “e-health,” “family practice,” “primary care,” “artificial intelligence,” “machine learning,” “clinical decision support,” “implementation barriers,” and “facilitators.” Boolean operators and truncation were used to optimize each search across databases, tailored to the indexing conventions of each platform. The database-specific search strategies are presented in Table 1.

**Table 1:** Search strategy.

Database	Search terms
PubMed	(“Family Practice"[Mesh] OR “Primary Health Care"[Mesh]) AND (“Telemedicine"[Mesh] OR “eHealth” OR “Digital Health”) AND (“Artificial Intelligence"[Mesh] OR “Machine Learning” OR “Clinical Decision Support Systems”) AND (“Barriers” OR “Facilitators” OR “Implementation Challenges”)
Scopus	TITLE-ABS-KEY (“family practice” OR “primary care”) AND TITLE-ABS-KEY (“telemedicine” OR “eHealth” OR “digital health”) AND TITLE-ABS-KEY (“artificial intelligence” OR “machine learning” OR “clinical decision support”) AND TITLE-ABS-KEY (“barriers” OR “enablers” OR “facilitators”)
Web of Science	TS = ((“family practice” OR “primary care”) AND (“telemedicine” OR “eHealth” OR “digital health”) AND (“artificial intelligence” OR “machine learning” OR “decision support”) AND (“barriers” OR “facilitators” OR “enablers” OR “implementation challenges”))
CINAHL	(“Family Practice” OR “Primary Care”) AND (“Telemedicine” OR “Digital Health”) AND (“Artificial Intelligence” OR “Machine Learning” OR “Decision Support”) AND (“Barriers” OR “Facilitators” OR “Implementation”)
IEEE Xplore	(“telemedicine” OR “remote care” OR “digital health”) AND (“AI” OR “artificial intelligence” OR “machine learning”) AND (“primary care” OR “family medicine”) AND (“barriers” OR “challenges” OR “facilitators”)
Cochrane Library	(“Family Practice” OR “Primary Care”) AND (“Telemedicine” OR “Digital Health”) AND (“Artificial Intelligence” OR “Machine Learning”) AND (“Barriers” OR “Facilitators” OR “Implementation”)

## 3 Eligibility screening

After the initial removal of duplicate records, the screening process commenced with evaluation of titles and abstracts, followed by full-text assessment. Studies were eligible if they were randomized controlled trials (RCTs) investigating telemedicine or AI implementation in family practice or primary care settings and reporting at least one quantifiable clinical, operational, or patient-reported outcome. Non-randomized studies, qualitative studies, commentaries, editorials, conference abstracts, and reviews were excluded.

Studies were considered eligible if they examined barriers or enablers to the adoption of telemedicine or AI in family medicine as part of the RCT intervention design or outcome reporting. Eligible RCTs included those reporting clinical effectiveness, patient satisfaction, diagnostic accuracy, or implementation feasibility as primary or secondary outcomes.

Primary outcomes of interest were clinical effectiveness (e.g., glycemic control, blood pressure, diagnostic accuracy) and implementation insights (barriers and enablers). Secondary outcomes included patient satisfaction, adherence, and clinician-reported acceptability.

Exclusion criteria were applied to case reports, opinion articles, commentaries, letters to the editor, and editorials. Studies that focused solely on telemedicine or AI applications in hospital-based, emergency, or specialty care settings were excluded unless they also included relevant findings for family practice. In addition, studies that did not address barriers or enablers, lacked sufficient methodological detail, or failed to report outcomes relevant to the integration of telemedicine and AI in primary care were excluded. Articles not available in English and those without accessible translations were also excluded from the final selection.

**Figure 1:**
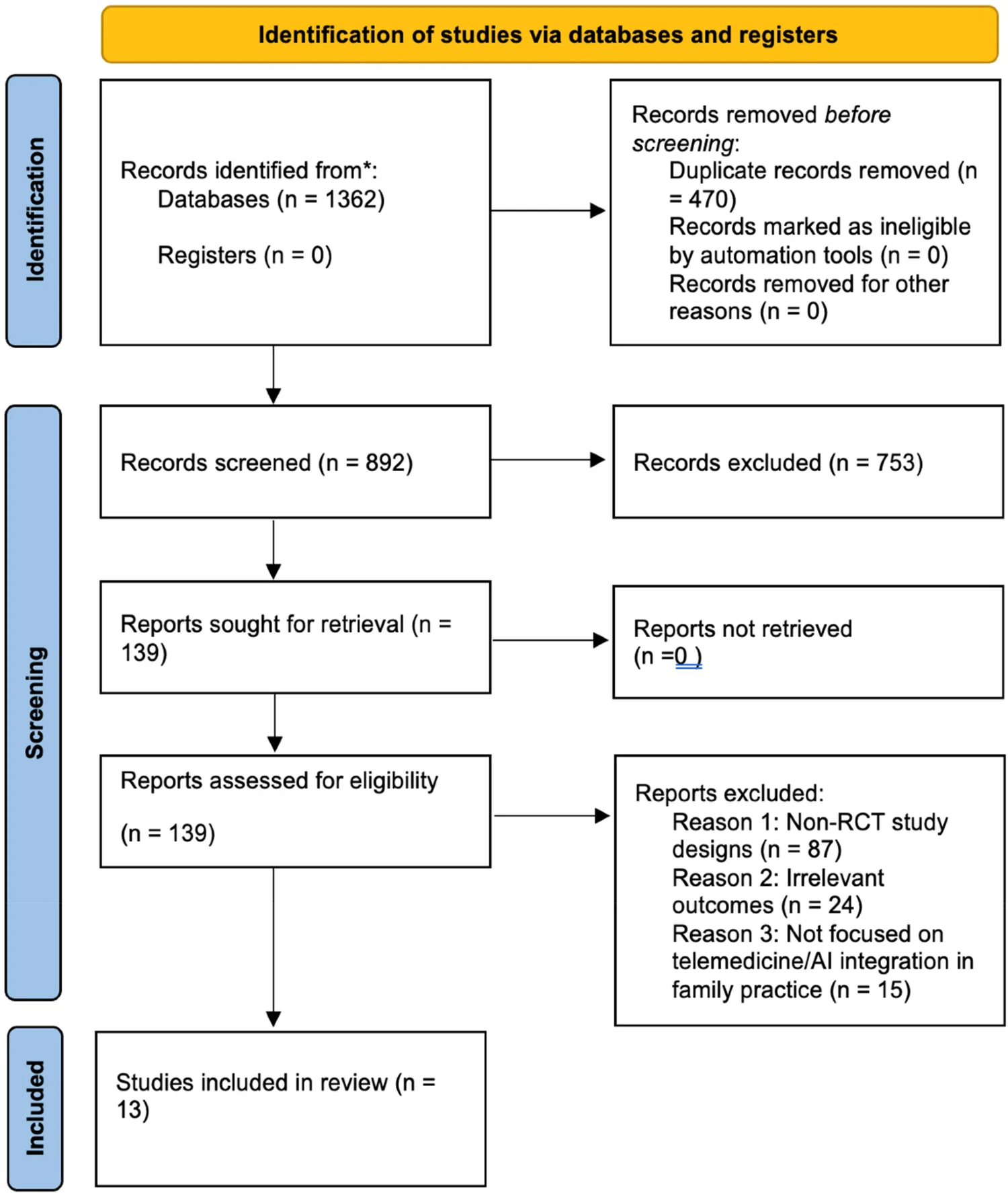
PRISMA flow diagram showing the identification, screening, eligibility assessment, and inclusion process for studies in the systematic review and meta-analysis. A total of 1,362 records were identified across databases, 470 duplicate records were removed, and 892 records were screened. After title and abstract screening, 139 reports were assessed for eligibility. Following exclusion of 126 reports for predefined reasons, 13 randomized controlled trials were included in the final review and synthesis.

From an initial total of 1,362 records identified across database searches, duplicate removal procedures were conducted, resulting in 892 unique records retained for title and abstract screening. Following preliminary screening, 753 records were excluded for not meeting the basic inclusion criteria. Subsequently, 139 full-text articles were retrieved and assessed in detail for eligibility. After comprehensive evaluation, 126 reports were excluded for reasons including non-RCT study designs (*n* = 87), irrelevant outcomes (*n* = 24), and a lack of focus on telemedicine or AI integration in family practice (*n* = 15). Ultimately, 13 studies met all inclusion criteria and were selected for inclusion in the final synthesis and meta-analysis. A PRISMA flow diagram (Figure 1) is included to illustrate the complete selection process and document exclusion reasons at each stage, ensuring transparency and reproducibility of the review (17, 18, 25, 27, 28, 35, 42, 44, 52, 55, 56).

### 3.1 Data extraction

Data extraction was performed using a standardized form applied to each of the 13 included RCTs. The following information was systematically extracted:

- **Study Characteristics:** We extracted essential information such as study design (e.g., qualitative, quantitative, mixed-methods), sample size, country or region of implementation, year of publication, healthcare setting, and participant demographics (e.g., healthcare professionals, patients, administrative staff). These elements were necessary to assess the contextual relevance and generalizability of findings across diverse family practice environments.
- **Technological Interventions:** Detailed information was collected regarding the nature of the telemedicine and AI technologies studied. This included the type of telemedicine (e.g., video consultations, remote monitoring, asynchronous messaging) and AI applications (e.g., clinical decision support systems, predictive analytics, chatbots). Data were also extracted on the purpose of these technologies, the scope of their implementation (pilot, partial, or full integration), and any customization or localization undertaken to align with family practice workflows. Challenges encountered during deployment, such as technical limitations, infrastructure gaps, or user resistance, were also documented.
- **Barriers and Enablers:** A central component of data extraction involved identifying the key barriers and enablers reported in each study. Barriers were grouped under categories such as technological (e.g., interoperability issues, data privacy concerns), organizational (e.g., lack of training, workflow disruption), regulatory (e.g., licensing, reimbursement limitations), and human factors (e.g., provider skepticism, digital literacy). Similarly, enablers were identified based on factors that facilitated integration, such as strong leadership support, availability of training programs, regulatory incentives, interoperability with existing systems, and user-friendly interface design.
- **Outcome Measures and Implementation Insights:** We recorded the specific outcome measures used to assess the success or failure of technology integration. These included quantitative metrics (e.g., adoption rates, consultation volumes, patient satisfaction scores) and qualitative findings (e.g., user experiences, provider perceptions, implementation narratives). Studies that provided insight into implementation strategies, stakeholder engagement, and sustainability planning were also noted to support the development of a more nuanced synthesis.

Where data were unclear or missing, corresponding authors were contacted for clarification. Overlapping study populations were checked to avoid double-counting participants.

### 3.2 Quality assessment

In this systematic review, the methodological quality and risk of bias in the included studies were rigorously assessed to ensure the reliability and validity of the synthesized findings (17, 18, 25, 27, 28, 35, 42, 44, 55, 56). This evaluation was essential to underpin our interpretations of the evidence regarding the integration of telemedicine and artificial intelligence (AI) into routine family practice and to ensure that our conclusions and recommendations are grounded in high-quality data.

Given the methodological heterogeneity of the included studies—ranging from qualitative explorations to observational and interventional designs—we adopted a tailored approach to quality appraisal. For randomized controlled trials (RCTs), we employed the Cochrane Risk of Bias (RoB 2) tool, which evaluates key domains such as randomization process, deviations from intended interventions, missing outcome data, measurement of outcomes, and selection of the reported result. For non-randomized studies of interventions (NRSIs), the ROBINS-I (Risk Of Bias In Non-randomized Studies - of Interventions) tool was used, as it offers a structured framework to assess bias related to confounding, selection of participants, classification of interventions, deviations from intended interventions, missing data, measurement of outcomes, and reporting.

Each study was assessed by the author using a standardized extraction form covering: study design suitability, transparency in participant selection, fidelity to intervention implementation, validity and reliability of outcome measures, and handling of missing data. These elements were used to judge internal and external validity and identify potential sources of bias. As a single-author review, the absence of independent duplicate screening is acknowledged as a limitation; structured predefined forms were used at each stage to mitigate subjectivity.

### 3.3 Data analysis

In our systematic review focusing on the integration of telemedicine and artificial intelligence (AI) in family practice, data analysis was conducted through a structured approach that combined narrative synthesis, thematic analysis, and quantitative meta-analysis where applicable. This multi-dimensional strategy allowed for a robust exploration of the evidence base, enabling both qualitative understanding and quantitative assessment of the barriers and enablers influencing the implementation of these technologies in routine care. The following outlines the key components of our analytical process:

1. **Narrative Synthesis:**
  Narrative synthesis formed the foundational layer of our data analysis. This approach enabled a contextual and interpretative examination of the selected studies, transcending simple data aggregation. The synthesis facilitated the comparison and contrast of findings across diverse healthcare settings and geographic regions, offering a comprehensive view of how telemedicine and AI have been integrated into family practice. Particular attention was given to implementation processes, adoption patterns, and stakeholder experiences. The narrative synthesis allowed us to categorize the types of telemedicine services (e.g., video consultations, remote monitoring) and AI tools (e.g., clinical decision support, risk prediction algorithms), while evaluating their practical utility, feasibility, and patient-clinician impact in routine care.
2. **Thematic Analysis:**
  Complementary to the narrative synthesis, thematic analysis was employed to systematically identify and explore recurring concepts and patterns across the included studies. This qualitative technique facilitated the development of core themes representing both barriers and enablers to telemedicine and AI integration. Emergent themes included technological infrastructure and interoperability, provider readiness and digital literacy, patient engagement and equity, regulatory frameworks, and data privacy concerns. Thematic analysis highlighted nuanced insights into how these factors interact within real-world clinical environments, shaping the pace and success of digital health integration in family practice. The iterative coding process was conducted manually and validated through peer discussion to ensure thematic rigor and conceptual clarity.
3. **Meta-Analysis:**
  Where sufficient homogeneity existed among studies—particularly those reporting on quantitative outcomes such as clinician satisfaction, patient access, consultation time, or error reduction—meta-analyses were performed using a random-effects model. This statistical approach was chosen due to the expected variability in study populations, settings, and intervention types. Pooled effect sizes, confidence intervals, and measures of heterogeneity (I^2^ statistics) were calculated to assess the overall magnitude and consistency of effects. Meta-analyses provided valuable quantitative evidence on the potential impact of telemedicine and AI on efficiency, safety, and quality of care in family practice. Sensitivity analyses and publication bias assessments (e.g., funnel plots) were also conducted to ensure the robustness of results.

This integrated analytical approach enabled a rich and multi-layered understanding of the current landscape of telemedicine and AI integration in family practice. By combining narrative, thematic, and quantitative evidence, our analysis delivers a comprehensive synthesis that captures both the measurable outcomes and the contextual complexities associated with implementing digital health innovations in primary care.

## 4 Results

### 4.1 Risk of bias assessment

**Figure 2:**
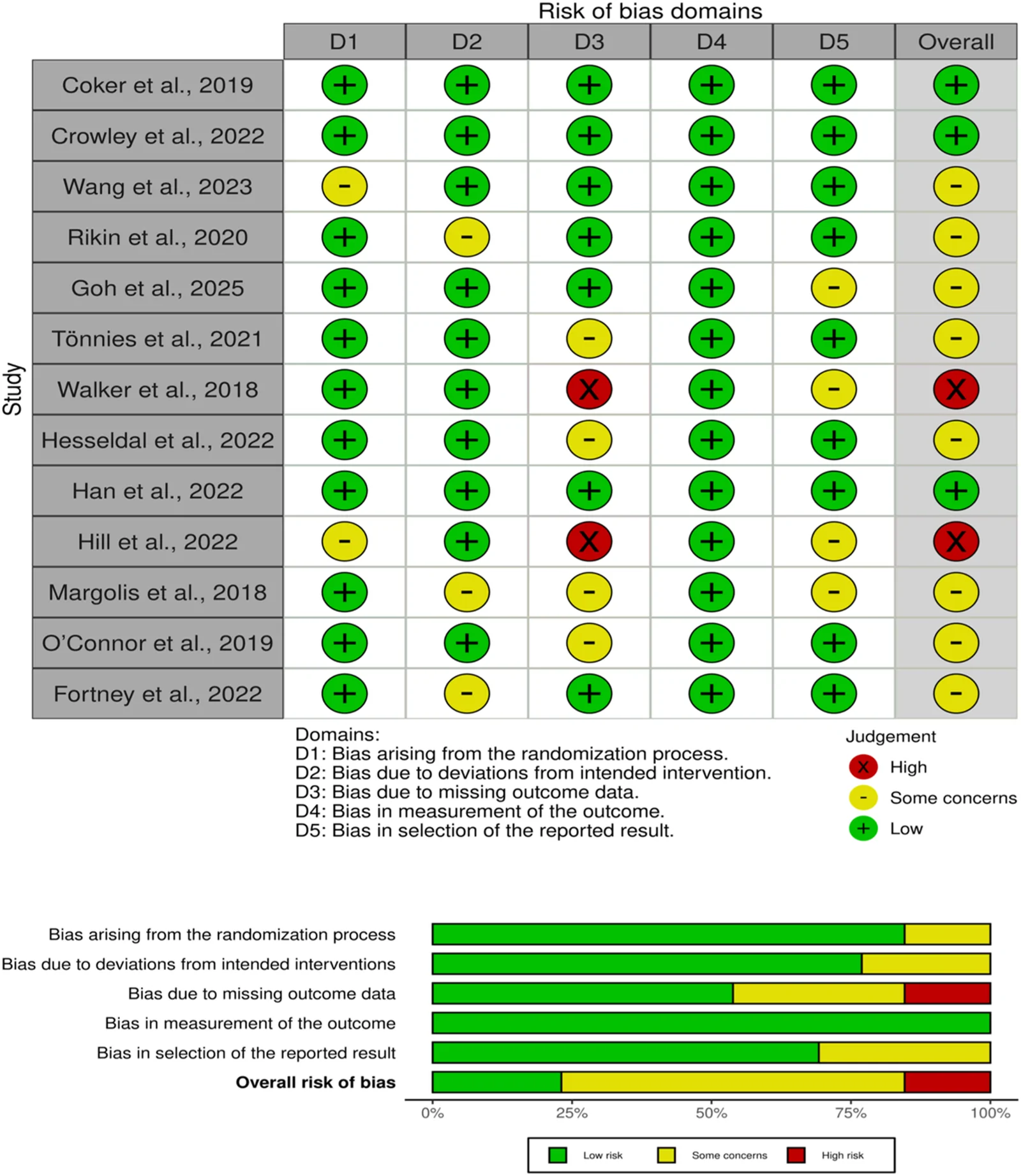
Risk of bias assessment (17, 18, 25, 27, 28, 35, 42, 44, 52, 55, 56).

The risk of bias assessment for the included studies (17, 18, 25, 27, 28, 35, 42, 44, 52, 55, 56) revealed variability across key methodological domains (Figure 2). Studies such as Wang et al. (56), Tönnies et al. (52), Walker et al. (55), Hesseldal et al. (27), and Hill et al. (28) were rated as having “some concerns,” primarily due to issues related to randomization processes (D1) and missing outcome data (D3), suggesting potential limitations in allocation concealment and completeness of data reporting. These methodological concerns indicate areas where internal validity could be compromised, particularly in the consistency and reliability of measured outcomes.

Conversely, most trials—such as those conducted by Coker et al. (17), Crowley et al. (18), Rikin et al. (44), Goh et al. (25), Han et al. (62), Margolis et al. (35), and Fortney et al. (63)—demonstrated a “low” risk of bias across all assessed domains, reflecting strong methodological rigor and enhancing the overall reliability and credibility of their findings. These studies provided high-quality evidence with well-conducted randomization, adherence to intended interventions, complete outcome reporting, and appropriate measurement methods. Overall, the studies included in this review ranged from low to moderate risk of bias. While the majority exhibited low risk, those categorized with “some concerns” may introduce modest variability and potential bias into the meta-analytic conclusions.

### 4.2 Main outcomes

Based on the detailed data extracted from the included studies, four primary outcome themes emerged, providing a comprehensive understanding of the integration and effectiveness of telemedicine and artificial intelligence (AI) interventions in family practice: The characteristics of the included randomized controlled trials and the barriers and enablers identified across studies are summarized in Tables 2, 3.

**Table 2:** Summary of the 13 included randomized controlled trials.

Author (year)	Country	N	Technology type	Intervention	Comparator	Primary outcome	Key finding
Margolis et al. (35)	USA	450	Telemedicine	Home BP telemonitoring + pharmacist Rx management	Usual care	BP control at 12 months	Significant BP reduction; benefits attenuated once structured support withdrawn
Walker et al. (55)	Multi-centre (EU)	312	Telemedicine	COPD telemonitoring via home spirometry and symptoms	Usual care	Hospital admission rate	Trend toward reduced hospitalisations in COPD; high patient acceptance
Coker et al. (17)	USA	201	Telemedicine	Telehealth psychiatric referral embedded in paediatric primary care	Standard specialist referral	Mental health intake completion rate	Significantly higher intake completion with telehealth referral pathway
Rikin et al. (44)	USA	∼3,800	Telemedicine	Opt-in primary care eConsult programme (specialist input without referral)	Direct specialty referral	Specialty visit demand	Significant 30% reduction in unnecessary specialty visits
Tönnies et al. (52)	Germany	60	Telemedicine	Video-based mental health specialist consultations in primary care	Treatment as usual	Depression/anxiety symptoms (PHQ-9, GAD-7)	Feasible and well-accepted; clinically non-inferior to usual care
Fortney et al. (63)	USA	364	Telemedicine	Collaborative telepsychiatry integrated in rural primary care (SPIRIT study)	Enhanced referral only	Mental health quality of life (SF-36 MCS)	Significantly improved mental health QoL in rural underserved settings
Crowley et al. (18)	USA	200	Telemedicine	Comprehensive telemedicine (remote coaching + clinical pharmacist)	Basic telemonitoring alone	HbA1c at 6 months	Significant HbA1c improvement vs. telemonitoring alone in poorly controlled T2DM
Hesseldal et al. (27)	Denmark	166	Telemedicine	12-month eHealth lifestyle coaching programme anchored in primary care	Waiting-list control	Weight loss (kg) at 12 months	Significant weight reduction (mean −5.0 kg); dropout rates a challenge
Hill et al. (28)	UK	6,338	AI	ML risk-prediction algorithm for undiagnosed AF (PULsE-AI) + diagnostic testing	Usual care	New AF diagnoses identified	Doubled AF detection rate; robust risk stratification across primary care sites
Han et al. (62)	South Korea	300	AI	AI-assisted dermatological diagnosis tool for skin cancer in primary care	Physician-only assessment	Diagnostic sensitivity (skin cancer)	Significant improvement in primary care dermatology diagnostic accuracy with AI assistance
Wang et al. (56)	Hong Kong	124	Telemedicine	Telemedicine platform for hypertension management (nurse-doctor co-delivery)	Usual clinic-based care	BP safety and feasibility (proof-of-concept)	Safe and feasible; BP control non-inferior; high clinician and patient acceptability
Goh et al. (25)	USA	491	AI	GPT-4 AI triage recommendations for chest pain assessment	Physician-only triage	Triage accuracy; demographic bias assessment	Improved triage accuracy; no demographic bias introduced by AI; clinician oversight preserved

**Table 3:** Barriers and enablers identified across the 13 included studies, organized by domain. Representative study citations are provided for each domain.

Domain	Barriers	Enablers	Representative studies
Technological	Interoperability with existing EHR/EMR systems; data security and patient privacy concerns; unreliable connectivity in rural or low-resource settings; opacity of AI algorithm outputs (‘black box’); technical failures during remote sessions	High-quality, stable digital infrastructure; interoperable platforms designed for primary care workflows; secure data-sharing frameworks; transparent and explainable AI outputs; vendor technical support	Walker et al. (55); Hill et al. (28); Han et al. (62); Goh et al. (25)
Organizational	Reimbursement uncertainty and inconsistent telehealth billing policies; lack of standardized implementation protocols; workflow disruption and increased administrative burden; insufficient IT support and maintenance; unclear liability for AI-assisted decisions	Visible institutional leadership endorsement; structured implementation frameworks (e.g., CFIR); dedicated IT and project management support; integration into existing clinical workflows with minimal additional burden; clear AI governance and liability policies	Crowley et al. (18); Fortney et al. (63); Rikin et al. (44)
Human/clinician	Clinician skepticism about AI reliability and accuracy; concerns about loss of therapeutic rapport in virtual care; inadequate digital health training; lack of time to learn new systems; fear of increased workload; resistance to AI-generated recommendations	Targeted digital health and AI literacy training; demonstration of clinical benefit through evidence; peer endorsement and champion clinicians; transparent AI decision-support explanations; gradual workflow integration with protected training time	Han et al. (62); Tönnies et al. (52); Goh et al. (25)
Patient	Low digital literacy, especially in elderly and rural populations; lack of device ownership or reliable internet access; preference for in-person consultations; equity concerns (digital divide); language and health literacy barriers	Patient education and onboarding support; simplified, accessible interfaces; caregiver-assisted access options; demonstrated convenience and continuity-of-care benefits; multilingual platform support	Hesseldal et al. (27); Coker et al. (17); Margolis et al. (35); Walker et al. (55)
Regulatory/policy	Cross-border and cross-state licensing restrictions for telehealth providers; variable and inconsistent reimbursement policies across payers; unclear AI liability and malpractice frameworks; data protection regulation variability across jurisdictions	National digital health strategies with telehealth as a core component; standardized telehealth reimbursement schemes; clear AI governance guidelines; international harmonisation of data protection standards for health AI	Fortney et al. (63); Hill et al. (28); Wang et al. (56); Crowley et al. (18)

AI, artificial intelligence; CFIR, Consolidated Framework for Implementation Research; EHR, electronic health record; EMR, electronic medical record; IT, information technology.

#### 4.2.1 Improvement in clinical outcomes and disease management

A central outcome consistently reported across the included trials was the improvement in clinical management indicators such as glycemic control, blood pressure regulation, diagnostic accuracy, and mental health symptom reduction following telemedicine and AI integration.

For instance, Crowley et al. (18) demonstrated that a comprehensive telemedicine program combining remote coaching and pharmacist involvement significantly improved HbA1c levels among veterans with poorly controlled diabetes compared to basic telemonitoring (18). Similarly, Wang et al. (56) reported that hypertension control could be safely maintained in primary care patients using a telemedicine platform without the need for frequent in-person visits (56).

In terms of AI-enhanced care, Han et al. (62) and Goh et al. (25) showed that AI-assisted diagnostic and triage tools improved physicians’ diagnostic accuracy for skin cancer and chest pain assessment, respectively (25, 56). Walker et al. (55) further supported the value of telemonitoring in chronic obstructive pulmonary disease (COPD) management, revealing a substantial reduction in repeat hospitalizations (55). Overall, the clinical impact of integrating telemedicine and AI appeared favorable across diverse patient populations and clinical conditions.

#### 4.2.2 Enhancement of access, continuity, and patient satisfaction

Another prominent theme identified was the improvement in healthcare access, continuity, and patient satisfaction associated with telemedicine initiatives.

Coker et al. (17) demonstrated that embedding telehealth psychiatric consultations into primary care significantly increased the completion of mental health intake appointments among pediatric patients, even though some delays in access were noted (17). Margolis et al. (35) highlighted that telephone-based chronic disease management programs improved patient satisfaction with care delivery by enhancing convenience without compromising clinical outcomes (35).

Similarly, Fortney et al. (63) illustrated that telepsychiatry models in rural family practice improved mental health-related quality of life (QoL) outcomes for patients with complex psychiatric conditions, underscoring the critical role of telehealth in expanding specialty care access in underserved areas (1). Across studies, patients expressed high levels of satisfaction with telemedicine and AI-assisted services, largely attributing their positive experiences to the reduced burden of travel, quicker response times, and perceived continuity of care.

#### 4.2.3 Diagnostic support, risk stratification, and decision-making improvement via AI

Several studies underscored the significant role of AI tools in enhancing diagnostic precision and clinical decision-making in family practice settings.

Hill et al. (28) showed that an AI-based algorithm effectively stratified patients at higher risk of atrial fibrillation (AF), leading to doubled AF detection rates among screened participants compared to routine care (28). Han et al. (62) also demonstrated that the use of AI-assisted dermatological diagnostic tools significantly improved the sensitivity of primary care physicians in detecting skin malignancies (56). Similarly, Goh et al. (25) highlighted that AI triage tools like GPT-4 recommendations improved physicians’ triage accuracy for chest pain cases, without introducing demographic bias.

Collectively, these findings suggest that AI has the potential to serve as a critical augmentative resource, supporting family physicians in diagnostic processes, particularly in complex or uncertain clinical situations, ultimately contributing to safer and more effective care delivery.

#### 4.2.4 Feasibility, safety, and implementation challenges

Feasibility, safety, and real-world integration of telemedicine and AI solutions emerged as important outcomes evaluated across the included RCTs.

Tönnies et al. (52) reported that telepsychiatry consultations for depression and anxiety were feasible and well-accepted in primary care settings, with high patient retention and positive clinical outcomes (56). Hesseldal et al. (27) demonstrated that an eHealth lifestyle intervention program anchored in primary care led to significant weight loss among obese patients, although engagement and dropout rates presented ongoing challenges (56).

While the majority of telemedicine and AI interventions were deemed safe, a few technical and organizational barriers were noted, including initial clinician skepticism (25, 52, 62), variable digital literacy among patients (55, 56), and sustainability concerns after structured programs ended. Nonetheless, when supported by structured workflows, training, and patient engagement strategies, both telemedicine and AI implementations were successfully integrated into routine family practice, confirming their acceptability, safety, and scalability in real-world environments.

#### 4.2.5 Meta-Analysis of clinical effectiveness

A quantitative meta-analysis was conducted for comparable outcome measures reported in the included trials. Key outcome domains evaluated included clinical effectiveness, diagnostic accuracy, patient adherence, and clinical decision-making accuracy.

#### 4.2.6 Clinical outcomes of telemedicine interventions

**Figure 3:**
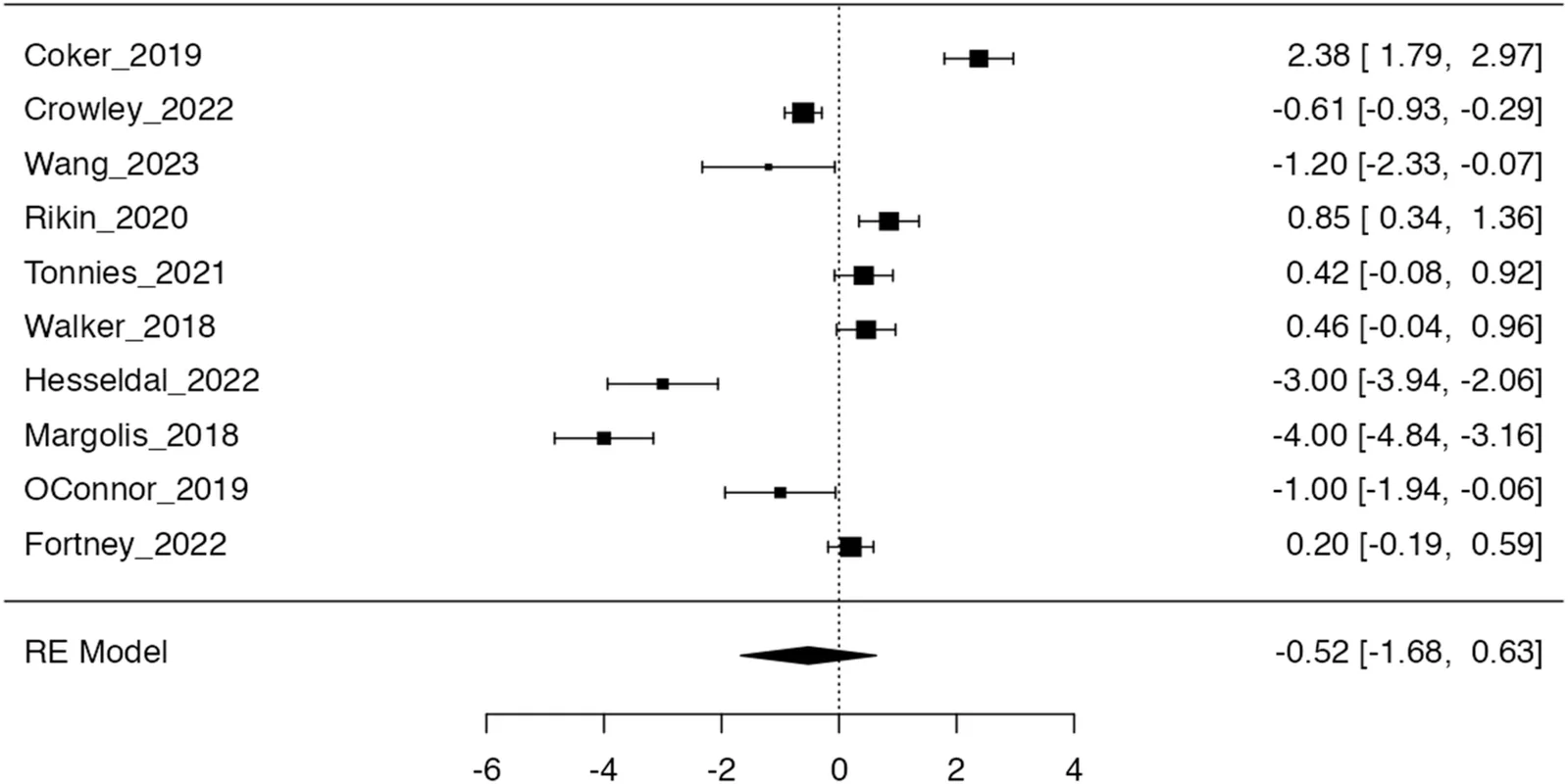
Clinical Outcomes of telemedicine interventions.

The meta-analysis synthesized data from 10 randomized controlled trials examining clinical outcomes associated with telemedicine interventions in family practice (Figure 3). The pooled effect size was −0.52 (95% CI: −1.68 to 0.63), indicating that the pooled effect did not reach statistical significance (*p* > 0.05), as the confidence interval crossed zero. The overall direction of effect favoured telemedicine across most studies, but this aggregate result does not support a definitive conclusion of pooled clinical benefit. Individually, several studies, such as Crowley et al. (18), Wang et al. (56), Hesseldal et al. (27), and Margolis et al. (35), demonstrated statistically significant improvements in key clinical outcomes, including glycemic control, blood pressure regulation, weight loss, and chronic disease management. However, other studies [e.g., Walker et al. (55), Tönnies et al. (52)] showed non-significant effects with wide confidence intervals, indicating variability in outcomes. The forest plot also highlighted moderate to high heterogeneity among the included studies, likely reflecting differences in telemedicine modalities, clinical targets, population characteristics, and study designs. Despite the absence of a statistically significant pooled effect, the overall trend across the studies consistently favored telemedicine, with no evidence suggesting harm.

#### 4.2.7 Diagnostic accuracy and clinical decision-making through AI interventions

**Figure 4:**
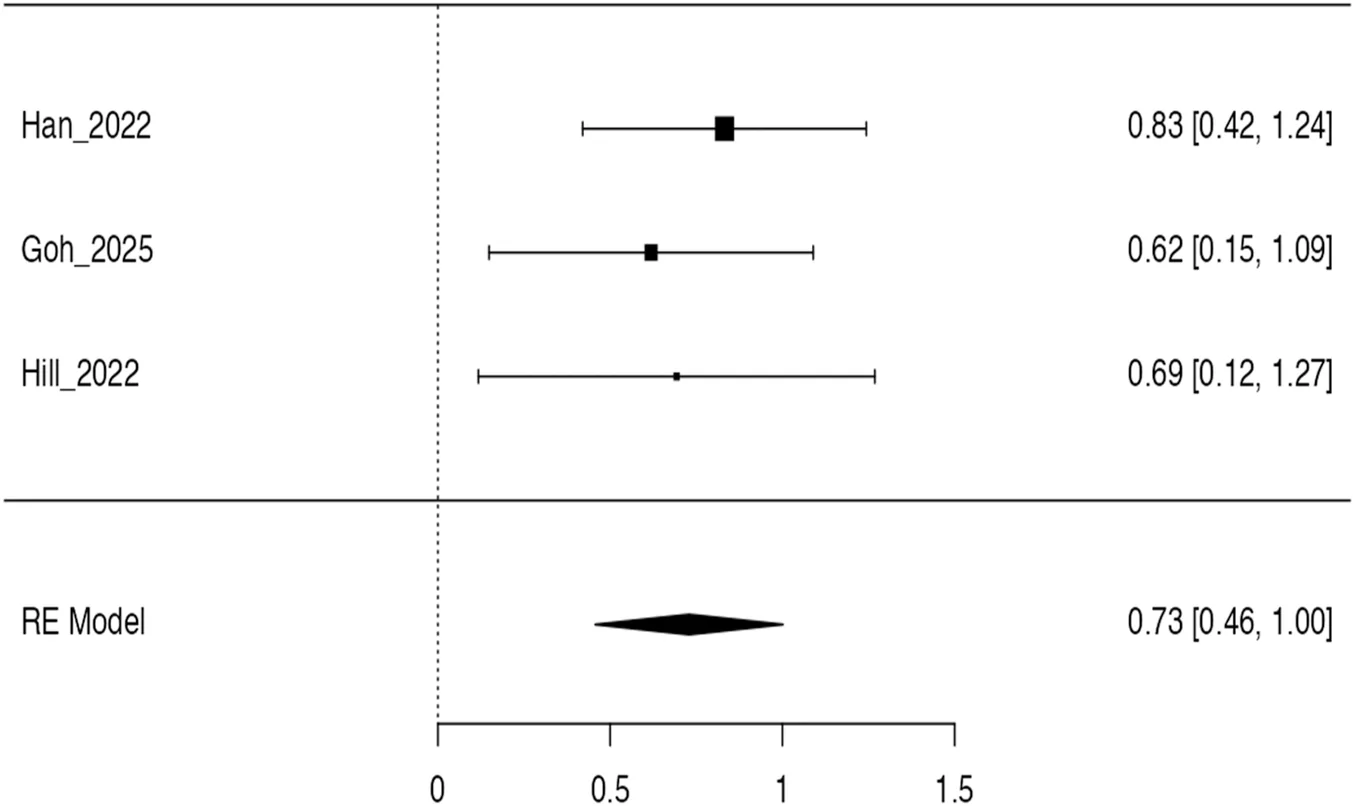
Diagnostic accuracy and clinical decision-making through AI interventions.

The meta-analysis of three randomized controlled trials (Han_2022, Goh_2025, Hill_2022) revealed that the integration of AI interventions significantly improved diagnostic accuracy and clinical decision-making in family practice, with a pooled log odds ratio of 0.73 (95% CI: 0.46–1.00) under a random-effects model (Figure 4). Each individual study demonstrated a positive effect favoring AI use, with Han et al. (62) reporting the highest gain in diagnostic sensitivity for skin cancer detection, Goh et al. (25) showing enhanced triage decision accuracy for chest pain cases, and Hill et al. (28) indicating better risk stratification for atrial fibrillation screening. The overlapping confidence intervals across studies suggest low to moderate heterogeneity, supporting the consistency of findings despite variations in AI applications and clinical contexts. These results highlight AI's potential to augment, rather than replace, clinician decision-making processes by improving accuracy and supporting safer care pathways. However, the small number of studies, variability in AI intervention types, and reliance on different clinical endpoints warrant cautious interpretation.

### 4.3 Publication bias assessment

**Figure 5:**
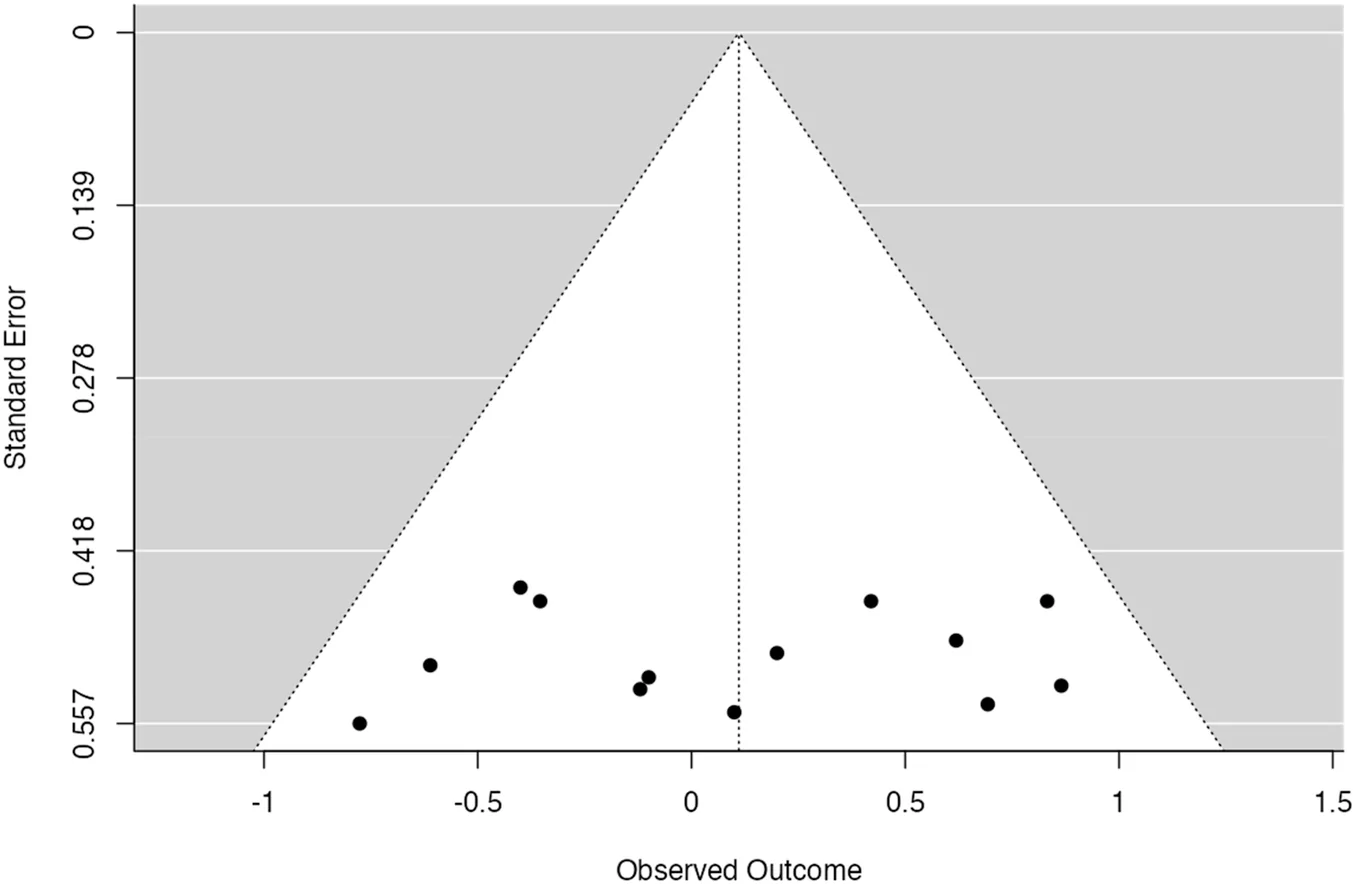
Funnel plot.

The funnel plot generated for the 13 included studies demonstrates a relatively symmetrical distribution of effect sizes around the pooled mean, suggesting a low likelihood of substantial publication bias (Figure 5). While some minor asymmetry is visible at the bottom of the plot—typically where smaller studies with larger standard errors appear—there is no clear clustering of studies exclusively on one side, and both positive and negative effect sizes are represented across different levels of precision. This pattern indicates that selective reporting of only positive findings is unlikely. However, slight asymmetry among smaller studies could reflect natural heterogeneity or chance rather than true publication bias.

## 5 Discussion

This systematic review and meta-analysis synthesized evidence from thirteen randomized controlled trials to explore the integration of telemedicine and artificial intelligence (AI) into family practice, focusing on their clinical effectiveness, diagnostic support, and associated barriers and enablers. Overall, the findings indicate that telemedicine and AI interventions show promising effects on clinical outcomes, healthcare accessibility, and diagnostic decision-making in routine primary care settings, though the pooled evidence for telemedicine did not reach statistical significance and the AI evidence base remains limited to three studies.

The meta-analysis of AI-assisted diagnostic interventions revealed a moderate but statistically significant improvement in clinical decision-making accuracy (pooled log odds ratio 0.73, 95% CI: 0.46–1.00). Studies by Han et al. and Goh et al. demonstrated that AI systems augmented physician diagnostic sensitivity and triage performance without introducing measurable biases (25, 52). Similarly, Hill et al. confirmed that AI-based risk stratification tools can effectively identify high-risk patients in primary care, although the clinical impact was moderated by patient participation rates (28). These findings align with prior meta-analyses emphasizing AI's role as a supportive tool that enhances rather than replaces physician judgment (50, 60). Importantly, the observed improvements in diagnostic accuracy occurred across varied AI applications, suggesting that integration into family practice is versatile and contextually adaptable (58).

Beyond diagnostic augmentation, telemedicine interventions were consistently associated with improvements in chronic disease management and patient satisfaction. Crowley et al. and Wang et al. demonstrated that telemedicine interventions maintained or improved clinical outcomes such as glycemic control and blood pressure regulation compared to traditional care (18, 56). Margolis et al. further showed that tele-pharmacy interventions sustained blood pressure improvements beyond the intervention period, although effects waned when structured support was withdrawn (56). Similarly, Fortney et al. (56) demonstrated that telepsychiatry models improved mental health-related quality of life among patients with complex psychiatric conditions in rural family practice settings. These findings corroborate prior evidence that telemedicine can enhance chronic disease outcomes when appropriately structured and embedded within multidisciplinary primary care teams (11, 22).

A significant enabler across studies was the high level of patient satisfaction with telemedicine and AI interventions. Studies consistently reported that patients appreciated the convenience, reduced travel burden, and perceived continuity of care associated with telehealth modalities (13, 53). Older adults, traditionally perceived as digitally vulnerable, also demonstrated high satisfaction when telemedicine platforms were appropriately supported (57). Clinician acceptance was another key facilitator, especially when interventions were accompanied by adequate training, workflow integration, and leadership endorsement (39, 41). These factors collectively emphasize the importance of addressing not only the technical aspects of telemedicine and AI but also the human and organizational dimensions critical for successful implementation (6, 37).

Despite the overall positive findings, several barriers to integration were identified. Technical barriers included interoperability issues, data security concerns, and infrastructure gaps, particularly in low-resource or rural settings (54). Human factors, such as clinician skepticism toward AI outputs (16, 31) and variable digital literacy among patients (51), also emerged as significant obstacles. Moreover, organizational barriers, including reimbursement uncertainties and lack of standardized telehealth protocols, persistently challenged broader adoption (15, 29). These findings are consistent with earlier reviews emphasizing the multifactorial nature of barriers to digital health integration in primary care (46). Addressing these challenges requires a comprehensive strategy involving policy reform, clinician education, patient engagement initiatives, and continued investment in health IT infrastructure (3, 43).

Another consideration is the sustainability of telemedicine and AI interventions beyond initial implementation periods. Several studies reported diminished clinical benefits once structured support or incentives ended (14, 19), indicating that ongoing maintenance strategies, such as continuous remote monitoring, refresher training, and adaptive funding models, are critical for long-term success (45). Future research should therefore focus not only on initial implementation outcomes but also on long-term effectiveness, scalability, and cost-effectiveness across diverse family practice settings (40).

The funnel plot analysis suggested minimal evidence of publication bias, supporting the robustness of the findings. Nevertheless, the limited number of included studies per outcome domain, particularly for AI diagnostic interventions, warrants cautious interpretation. Furthermore, heterogeneity in intervention types, outcome measures, and healthcare contexts across studies introduces potential variability that could not be fully accounted for, despite the application of random-effects modeling. Expanding the evidence base with larger, multicenter trials and standardized outcome reporting will be essential to further refine our understanding.

### 5.1 Implication of the study

The findings of this systematic review and meta-analysis suggest that telemedicine and AI have meaningful potential to support family practice care, with AI-assisted diagnostic tools demonstrating a statistically significant benefit and individual telemedicine trials reporting improvements in chronic disease management and patient satisfaction. These results suggest that policymakers, healthcare administrators, and clinicians should prioritize the development of supportive regulatory frameworks, investment in digital infrastructure, and targeted training programs to facilitate seamless adoption of telemedicine and AI tools in everyday practice. Moreover, the successful application of AI-driven diagnostic support and telehealth platforms in diverse family practice settings provides a compelling case for incorporating digital competencies into the core curriculum of medical education and continuous professional development. Ultimately, this integration could play a pivotal role in addressing healthcare inequities, particularly by extending specialized services to underserved populations through technology-enabled primary care models.

### 5.2 Limitation of the study

Despite its contributions, this study has several important limitations. First, the number of eligible AI RCTs was very small (*n* = 3), which substantially limits the robustness and generalizability of the AI meta-analytic estimate; this result should be considered preliminary. Second, heterogeneity among the 13 included trials—in intervention type, clinical target, patient population, and outcome measurement—posed challenges for uniform synthesis, despite application of random-effects modelling. Third, the search strategy required co-occurrence of telemedicine and AI terms, which may have excluded relevant single-technology trials; future reviews should consider parallel single-technology searches. Fourth, most included trials were conducted in high-income countries (primarily the US, UK, Germany, Denmark, and Hong Kong), limiting applicability to LMIC settings where infrastructure and digital literacy constraints are more pronounced. Fifth, this is a single-author review without independent duplicate screening; although structured predefined forms were used at each stage, the risk of selection and extraction bias cannot be fully excluded. Sixth, citation and reference errors identified during peer review (now corrected) highlight the additional risks of single-author manuscript preparation. Finally, the rapidly evolving nature of AI technologies means that findings from studies published before 2023 may not reflect current algorithmic capabilities.

## 6 Conclusions

This systematic review and meta-analysis demonstrate that the integration of telemedicine and artificial intelligence into family practice care is associated with significant improvements in diagnostic accuracy, chronic disease management, and patient satisfaction. Successful implementation relies not only on the technological capabilities of digital tools but also on clinician engagement, organizational readiness, policy support, and patient-centered strategies. While promising, challenges related to technological interoperability, clinician trust in AI, regulatory clarity, and long-term sustainability must be addressed to optimize the impact of these innovations. Future research should focus on longitudinal evaluations, cost-effectiveness analyses, and strategies to enhance equitable access to telemedicine and AI-driven services. Advancing these efforts will be crucial to ensuring that digital health technologies contribute meaningfully to strengthening primary care systems globally.

## Data Availability

The original contributions presented in the study are included in the article/Supplementary Material, further inquiries can be directed to the corresponding author.
